# ECPELLA as a bridge-to-decision in refractory cardiogenic shock: a single-centre experience

**DOI:** 10.1007/s12471-024-01872-w

**Published:** 2024-05-07

**Authors:** Jan-Willem Balder, Mariusz K. Szymanski, Linda W. van Laake, Pim van der Harst, Christiaan L. Meuwese, Faiz Z. Ramjankhan, Manon G. van der Meer, Jeannine A. J. M. Hermens, Michiel Voskuil, Eric E. C. de Waal, Dirk W. Donker, Marish I. F. J. Oerlemans, Adriaan O. Kraaijeveld

**Affiliations:** 1 Department of Cardiology, University Medical Centre Utrecht, Utrecht, The Netherlands; 2 Department of Cardiology, Erasmus Medical Centre, Rotterdam, The Netherlands; 3 Department of Intensive Care, Erasmus Medical Centre, Rotterdam, The Netherlands; 4 Department of Cardiothoracic Surgery, University Medical Centre Utrecht, Utrecht, The Netherlands; 5 Department of Intensive Care, University Medical Centre Utrecht, Utrecht, The Netherlands; 6 Department of Anaesthesiology, University Medical Centre Utrecht, Utrecht, The Netherlands; 7 Cardiovascular and Respiratory Physiology, Tech Med Centre, University of Twente, Enschede, The Netherlands

**Keywords:** Cardiogenic shock, Venoarterial extracorporeal membrane oxygenation, Impella, Observational study, Left ventricular assist device

## Abstract

**Background:**

In refractory cardiogenic shock, temporary mechanical support (tMCS) may be crucial for maintaining tissue perfusion and oxygen delivery. tMCS can serve as a bridge-to-decision to assess eligibility for left ventricular assist device (LVAD) implantation or heart transplantation, or as a bridge-to-recovery. ECPELLA is a novel tMCS configuration combining venoarterial extracorporeal membrane oxygenation with Impella. The present study presents the clinical parameters, outcomes, and complications of patients supported with ECPELLA.

**Methods:**

All patients supported with ECPELLA at University Medical Centre Utrecht between December 2020 and August 2023 were included. The primary outcome was 30-day mortality, and secondary outcomes were LVAD implantation/heart transplantation and safety outcomes.

**Results:**

Twenty patients with an average age of 51 years, and of whom 70% were males, were included. Causes of cardiogenic shock were acute heart failure (due to acute coronary syndrome, myocarditis, or after cardiac surgery) or chronic heart failure, respectively 70 and 30% of cases. The median duration of ECPELLA support was 164 h (interquartile range 98–210). In 50% of cases, a permanent LVAD was implanted. Cardiac recovery within 30 days was seen in 30% of cases and 30-day mortality rate was 20%. ECPELLA support was associated with major bleeding (40%), haemolysis (25%), vascular complications (30%), kidney failure requiring replacement therapy (50%), and Impella failure requiring extraction (15%).

**Conclusion:**

ECPELLA can be successfully used as a bridge to LVAD implantation or as a bridge-to-recovery in patients with refractory cardiogenic shock. Despite a significant number of complications, 30-day mortality was lower than observed in previous cohorts.

**Supplementary Information:**

The online version of this article (10.1007/s12471-024-01872-w) contains supplementary material, which is available to authorized users.

## What’s new?

- In refractory cardiogenic shock, temporary mechanical circulatory support (tMCS) can be used to improve tissue perfusion and oxygen delivery.
- tMCS could serve as a bridge-to-recovery or as a bridge-to-decision (improving current organ function and assessing contraindications for heart transplantation or permanent left ventricular assist device [LVAD] implantation).
- In recent years, venoarterial extracorporeal membrane oxygenation has been used in combination with Impella (ECPELLA) in patients with severe shock.
- In our cohort, patients supported with ECPELLA had a 30-day mortality of 20%, which is lower than reported in the literature; 50% underwent LVAD implantation.
- ECPELLA support is associated with important complications (bleeding, haemolysis and vascular complications).

## Background

Cardiogenic shock is characterised by low cardiac output and high filling pressures, leading to systemic hypoperfusion and pulmonary congestion. Cardiogenic shock can be classified as acute (due to, for example, acute coronary syndrome or myocarditis) or as acute-on-chronic (shock in patients with chronic heart failure) [1]. In order to maintain adequate tissue perfusion and oxygen delivery, (percutaneous) venoarterial extracorporeal membrane oxygenation (VA-ECMO) therapy can be applied, which improves systemic blood flow and gas exchange. However, VA-ECMO increases myocardial workload and decreases myocardial perfusion, potentially leading to further complications in an already failing heart [2]. Therefore, LV unloading strategies, such as the intra-aortic balloon pump (IABP) or Impella (Abiomed, Danvers, MA, USA), are necessary [3]. Based on cardiovascular computer simulations and observational data, IABP decreases afterload and intracardiac pressure slightly [4, 5]. Impella can ensure significant LV unloading and improve outcomes [6, 7]. Studies suggest that the use of Impella in combination with VA-ECMO (known as ECPELLLA) results in improved 30-day mortality [8].

Our tertiary centre is specialised in the management of patients with advanced heart failure, including permanent left ventricular assist device (LVAD) implantations and heart transplantations. These advanced therapies require careful selection of patients after a period of extensive screening, but long-term survival of patients receiving advanced heart failure therapy is good [9, 10]. We have been utilising temporary mechanical circulatory support with VA-ECMO as a bridge-to-decision, meaning temporary support to improve current organ function and to assess any existing contraindications for advanced heart failure therapies [11], or as a bridge-to-recovery. Since 2020, in selected cases, we have been using ECPELLA in refractory cardiogenic shock patients to further improve circulatory support and organ perfusion, which may extend the wait time to recovery or the optimal timing of LVAD implantation. In this study, we report our 2‑year experience with ECPELLA.

## Methods

### Study population

All consecutive patients who were supported with ECPELLA for refractory cardiogenic shock were included. Patients were classified using the Society of Cardiovascular Angiography and Intervention (SCAI) shock stage [12]. The decision to provide ECPELLA support was made by a multidisciplinary team comprising a cardiologist specialised in advanced heart failure, an intensivist, a cardiothoracic surgeon and cardiac anesthesiologist (and if necessary an interventional cardiologist). Patients supported between December 2020 and August 2023 were enrolled. Active LV unloading with Impella in combination with VA-ECMO can be a primary treatment, but Impella placement can also be considered as a bailout strategy. When a bailout strategy was used, we differentiated between early (< 2 h) and delayed (> 2 h) addition of Impella to VA-ECMO, or vice versa [13]. The requirement for informed consent was waived by the primary ethics committee, as this was a retrospective analysis.

### Mechanical support

The decision to perform temporary mechanical support is made by a multidisciplinary team. We do not have a pre-established protocol. LV unloading is not standard in VA-ECMO and when LV unloading is clearly necessary an Impella device is inserted. In case of doubt an IABP is more likely to be chosen. Decisions regarding mechanical support are based on clinical parameters (pulmonary oedema, hypoxaemia and end-organ perfusion), echocardiographic parameters (mainly severity of LV systolic failure, severity of LV dilatation and, in some cases, the aortic valve not opening) and invasive measurements (especially cardiac output and wedge pressure).

### Outcomes

The primary outcome was 30-day mortality. Secondary outcomes were 90-day mortality, LVAD implantation, cardiac recovery, and safety outcomes. Safety outcomes included thrombolysis in myocardial infarction (TIMI) major bleeding (i.e. intracranial bleeding or drop in haemoglobin [Hb] > 3.0 mmol/l), haemolysis (lactate dehydrogenase ≥ 1000 U/l in combination with two separate haptoglobin levels < 0.3 g/l, as previously described [8]), intervention because of access-site-related ischaemia, failure of mechanical support devices requiring extraction, and renal replacement therapy. Vascular access complications were stratified as ischaemic (limb ischaemia requiring intervention) or bleeding (requiring intervention).

### Statistical analysis

IBM SPSS Statistics, version 27 (IBM Corp., Armonk, NY, USA) was used for statistical analysis. Normally distributed variables were presented as mean with standard deviation, while non-normally distributed variables were presented as median and interquartile range.

## Results

### Baseline characteristics

Twenty patients were included. Tab. 1 shows patient characteristics at baseline; the average age of the participants was 51 years, and 70% of the patients were male. The majority of the patients (75%) were of Caucasian descent, 30% were current or previous smokers, and 15% of patients had diabetes mellitus. Table S1 (Electronic Supplementary Material) shows a detailed clinical profile for each patient.

**Table 1:** Baseline characteristics

Baseline characteristics	
Age (years, mean ± SD)	51.3 (9.4)
Gender (male, %)	14 (70)
BMI (kg/m^2^, mean ± SD)	24.7 (3.6)
Race (Caucasian, %)	15 (75)
Hypertension (*n*, %)	5 (25)
Diabetes mellitus (*n*, %)	3 (15)
Peripheral artery disease (*n*, %)	0 (0)
Ischaemic CVA (*n*, %)	1 (5)
Myocardial infarction (*n*, %)	3 (15)
Percutaneous revascularisation (*n*, %)	3 (15)
CABG (*n*, %)	0 (0)
Smoking (*n*, %)	
– Never smoked	13 (65)
– Previous smoker	3 (15)
– Current smoker	3 (15)
– Unknown	1 (5)
Atrial fibrillation (*n*, %)	3 (15)
Chronic kidney disease (*n*, %)	1 (5)
ICD (*n*, %)	5 (25)
Pacemaker (*n*, %)	1 (5)
Number of platelet aggregation inhibitors (*n*, %)	
– 0	11 (55)
– 1	2 (10)
– 2	7 (35)

*BMI* body mass index, *CVA* cerebrovascular accident, *CABG* coronary artery bypass graft, *ICD* implantable cardioverter-defibrillator

### Clinical profile of cardiogenic shock

The patients were in a state of refractory cardiogenic shock, with a mean heart rate of 109 bpm, low systolic (86 mm Hg) and diastolic (54 mm Hg) blood pressure (Tab. 2). Median lactate level was 3.0 mmol/l and mean creatinine level was 142 µmol/l. In most patients, inotropes (70%) and/or vasopressors (80%) were given. Before ECPELLA implantation, 25% of the patients had a cardiac arrest. Twelve patients (60%) were classified as SCAI shock stage D and seven (35%) as stage E.

**Table 2:** Clinical profile of cardiogenic shock during ECPELLA

Clinical profile of cardiogenic shock	
Heart rate (beats/min, mean ± SD)	109 (26)
Systolic blood pressure (mm Hg, mean ± SD)	86 (13)
Diastolic blood pressure (mm Hg, mean ± SD)	54 (13)
Rhythm	
– Sinus rhythm (*n*, %)	16 (80)
– Atrial fibrillation/atrial flutter (*n*, %)	4 (20)
Creatinine (µmol/l, mean ± SD)	142 (43)
Sodium (mmol/l, mean ± SD)	138 (5)
Potassium (mmol/l, mean ± SD)	4.2 (0.7)
Urea (mmol/l, mean ± SD)	15.7 (8.5)
Haemoglobin (mmol/l, mean ± SD)	8.2 (1.7)
Leucocytes (× 10^9^/l, mean ± SD)	20.8 (9.6)
Lactate (mmol/l, median/IQR)	3.0 (2.5–4.3)
Bilirubin (µmol/l, median/IQR)	13 (11–20)
CPR (mg/dl, median/IQR)	65 (11–149)
Thrombocytes (× 10^9^/l, median/IQR)	215 (146–270)
Inotropes use (*n*, %)	14 (70)
Vasopressor use (*n*, %)	16 (80)
Mechanical ventilation (*n*, %)	14 (70)
Previous IABP (*n*, %)	3 (15)
CPR before ECPELLA (*n*, %)	5 (25)
Cause of cardiogenic shock (*n*, %)	
– Myocarditis	3 (15)
– Acute coronary syndrome	8 (40)
– Acute failure with known cardiomyopathy	6 (30)
– Post-thoracotomy	2 (10)
– Pheochromocytoma	1 (5)
SCAI shock classification (*n*, %)	
– Stage C	1 (5)
– Stage D	12 (60)
– Stage E	7 (35)
ECPELLA indication (*n*, %)	
– Severe LV dysfunction/shock	14 (70)
– LV unloading	5 (25)
– Shock and high-risk PCI	1 (5)
Timing of ECPELLA support (*n*, %)	
– Simultaneous insertion	12 (60)
– Impella first ‘early’	2 (5)
– Impella first ‘late’	1 (5)
– VA-ECMO first ‘late’	5 (25)
Access site, left femoral artery (*n*, %)	12 (60)
Duration of ECPELLA support (h)	164 (98–210)

*ECPELLA* venoarterial extracorporeal membrane oxygenation combined with Impella, *IQR* interquartile range, *CPR* cardiopulmonary resuscitation, *IABP* intra-aortic balloon pump, *SCAI* Society of Cardiovascular Angiography and Intervention, *LV* left ventricular, *PCI* percutaneous cardiac intervention, *VA-ECMO* venoarterial extracorporeal membrane oxygenation

Acute heart failure (heart failure de novo) due to acute coronary syndrome was the cause of refractory cardiogenic shock in 45%, myocarditis in 15%, and one patient had a pheochromocytoma. Two patients needed haemodynamic support after thoracotomy. One of these patients had acute primary graft dysfunction after heart transplantation, and the other underwent a Bentall procedure which was complicated by occlusion of the left coronary artery due to thrombus (Tab. 2). Cardiogenic shock due to chronic heart failure was present in 30%.

Twelve patients (60%) received VA-ECMO and Impella support simultaneously, the main reasons being the severity of LV dysfunction and the expected need for LV unloading (Table S1). In five cases, VA-ECMO was used as the primary strategy, but because of insufficient LV unloading an Impella device was added, in all cases as a delayed strategy (> 2 h difference between VA-ECMO and Impella insertion). The main reasons were a combination of pulmonary oedema, LV dilatation and high wedge pressure. Interestingly, in three of these patients LV unloading with IABP was insufficient. Impella was the primary strategy in three patients, and early escalation to ECPELLA was performed because of ongoing cardiogenic shock in two patients, so mainly because of haemodynamic parameters. The median duration of ECPELLA support was 164 h (interquartile range 98–210).

### Clinical outcomes

The 30-day mortality rate was 20% (Tab. 3). The patients who died did not receive an LVAD, the contraindications being ongoing sepsis, poor neurological outcome after cardiac arrest and multiorgan failure. Three of the four patients who died were classified as SCAI cardiogenic shock stage E. LVAD implantation was performed in 50%, while urgent heart transplantation was never performed. In our study ECPELLA was used as a bridge-to-decision in 70% of the patients. Only 6 out of 20 patients (30%) were successfully weaned from ECPELLA after 30 days. In five patients a delayed strategy was chosen, one of those patients died within 30 days. Ninety-day mortality was 33%.

**Table 3:** Clinical outcomes, safety and complications of ECPELLA support

Clinical outcomes, safety and complications	
*Clinical outcomes*	
LVAD implantation (*n*, %)	10 (50)
Heart transplantation (*n*, %)	0 (0)
30-day cardiac recovery (*n*, %)	6 (30)
30-day mortality (*n*, %)	4 (20)
90-day mortality (*n*, %)	6 (33)
*Safety/complications*	
(i)CVA (*n*, %)	0 (0)
Vascular (*n*, %)	6 (30)
– Bleeding, requiring intervention	3 (15)
– Ischaemic, requiring intervention	3 (15)
– Dissection	1 (5)
Haemolysis (*n*, %)	5 (25)
Major bleeding (*n*, %)	8 (40)
– Vascular access site	2 (10)
– Gastrointestinal	2 (10)
– Haemothorax	2 (10)
– Pulmonary	1 (5)
Kidney failure requiring replacement therapy (*n*, %)	10 (50)
Impella failure requiring extraction (*n*, %)	3 (15)
Mitral valve replacement due to papillary rupture	1 (5)

*ECPELLA* venoarterial extracorporeal membrane oxygenation combined with Impella, *LVAD* left ventricular assist device, *(i)CVA* (ischaemic) cerebrovascular accident

### Safety/complications

As shown in Tab. 3, ECPELLA support was associated with major bleeding (40%). Two post-cardiotomy patients suffered from haemothorax and underwent re-thoracotomy several times within days after insertion. Two patients suffered from gastro-intestinal bleeding and one patient had repetitive pulmonary haemorrhages. Three patients had an Hb drop > 3 mmol/l without clear cause. One patient had a severe bleed during removal of the VA-ECMO. Of the patients with dual antiplatelet therapy, 29% had a major bleeding episode, and in patients without antiplatelet therapy the rate was 36% (data not shown). Haemolysis was observed in 25% cases, but in only one case was haemolysis thought to be so severe that Impella extraction was deemed necessary. Vascular access site complications occurred in 30%, with acute limb ischaemia in 15% and bleeding at the insertion site in 15% (two major bleeds).

In three cases, the Impella device was extracted due to complications. In one case, bleeding complications were so severe that removing the device was necessary, and an IABP was inserted instead. In another case, dislocation of the Impella device occurred after transferring the patient immediately following the procedure. In the last case, Impella dysfunction occurred due to thrombosis despite adequate positioning, high flow and adequate anticoagulation.

## Discussion

This retrospective study describes our first experience with ECPELLA support in refractory cardiogenic shock and shows some interesting findings. First, despite maximal pharmacological and mechanical support, 30-day mortality rate in our patients with refractory cardiogenic shock was 20%. Second, in our centre, ECPELLA was primarily utilised as a bridge-to-decision strategy. While awaiting cardiac recovery and end organ function, a multidisciplinary decision was made on potential exit strategies, which could be either the implantation of long-term LVAD support, cardiac transplant or palliative care. Cardiac recovery was seen in only 6 out of 20 patients (bridge-to-recovery) and in 50% an LVAD was implanted. ECPELLA support is of added value in a hospital with the potential to implant LVADs (or perform heart transplantation). Finally, our study highlights that the use of ECPELLA is associated with a notable occurrence of complications, such as major bleeding in 40% of cases, Impella failure requiring extraction in 15% of cases, and haemolysis in 25% of cases. It is important to consider these potential complications when evaluating the use of ECPELLA as a treatment option for patients with refractory cardiogenic shock.

### Thirty-day mortality

In our cohort, the observed 30-day mortality rate was lower than that reported in prior study cohorts. Previous small retrospective studies showed a 30-day mortality rate of 39 and 45% [14, 15], while a larger multicentre cohort study with 337 participants reported a 30-day mortality rate of 60% [8]. Interestingly, our 90-day mortality rate is 33%. The heterogeneity in study populations makes it challenging to compare these cohorts. In most studies, the primary underlying cause of cardiogenic shock was acute myocardial infarction, but in our study it occurred in only 45% of cases. Besides, it is plausible to assume that our tertiary referral hospital for advanced heart failure attracts patients with less severe cardiogenic shock with mono-organ failure who seems more likely to be candidates for advanced heart failure therapy. Furthermore, the availability of advanced heart failure therapies such as LVAD implantation and heart transplantation at our centre may have contributed to lower mortality rates. In fact, we performed many more LVAD implantations or heart transplantations compared to Iannaconne et al. (11% vs 50%) [7]. Our study cohort is too small to build mortality prediction models, but three of the four patients who died were classified as SCAI cardiogenic shock stage E. This raises the question whether ECPELLA support is truly helpful in those patients.

### Bridge-to-decision

The total number of LVAD implantations in this group is indeed remarkable. Urgent heart transplantation is infrequent in the Netherlands due to a shortage of organ donors. Consequently, LVAD implantation is the preferred option for long-term survival in cases of cardiogenic shock, either as a destination therapy or bridge to heart transplantation. Before proceeding to LVAD implantation, several contraindications need to be evaluated, including severe right ventricular dysfunction, active systemic infection, irreversible brain damage, active bleeding, contraindications for anticoagulation, and comorbidities with a poor prognosis. Moreover, sometimes surgical correction is necessitated owing to the presence of aortic or tricuspid regurgitation. Consideration of all these factors requires time, which is limited in cardiogenic shock. In such cases, ECPELLA can be used as a bridge-to-decision. Once approved for LVAD implantation, the long-term survival rate is good (88% 1‑year survival), especially with the latest HeartMate III LVADs (Abbott, St. Paul, MN, USA) [9, 16, 17], although this is lower in LVAD implantations after temporary mechanical support.

### Complications

As previously demonstrated, providing mechanical circulatory support with the Impella device in combination with VA-ECMO involves important complications. Compared to VA-ECMO, ECPELLA support is associated with a higher bleeding rate (21% vs 33%) [7], which is slightly lower compared to our observed bleeding rate of 40%. VA-ECMO is associated with a high bleeding rate due to the need for large-bore arterial and venous access, causing platelet dysfunction, and requiring anticoagulation. Adding an Impella device to VA-ECMO requires an additional large-bore arterial access. However, bleeding at the access site can be minimised by using ultrasound-guided and fluoroscopy-guided insertion and proper fixation of the Impella device at the insertion site. In addition to bleeding at the insertion site, we also encountered gastrointestinal and pulmonary bleeding, which could be due to acquired Von Willebrand syndrome. In the two post-cardiotomy patients, multiple thoracotomies were necessary because of bleeding complications, which could suggest restrained use of ECPELLA in those patients.

Another known complication of ECPELLA support is haemolysis, which is caused by the high shear stress on the erythrocytes due to the small inlet and outlet of the Impella device [6]. Haemolysis rates are reported to be between 22 and 45% [8, 14, 18, 19], which is comparable to our finding of 25%.

Finally, studies have shown a high rate of kidney replacement therapy in patients supported with ECPELLA. In our study, this was the case in 50% of patients, and a meta-analysis of five observational studies comparing VA-ECMO to ECPELLA patients showed a 50% rate of kidney replacement therapy as well. Interestingly, this was significantly higher compared to VA-ECMO patients (30%) [20]. This finding could be explained by the fact that patients with ECPELLA are more likely to survive, but other contributing factors, such as the cytotoxic effect of haemolysis, may also play a role.

### Limitations

Our study findings should be interpreted in light of several important considerations. Firstly, this is a retrospective observational study conducted at a single centre, and therefore generalising our results to other healthcare facilities is possible only to a limited extent. Additionally, our study population is highly specific due to the tertiary care for heart failure and access to LVAD implantation and heart transplantation. For instance, the primary cause of cardiogenic shock in most studies is acute myocardial infarction, but in our study this was not the case. Secondly, at the start of the study Impella devices were relatively new, and complications may have been overestimated due to the learning curve involved in using the device. Thirdly, given the heterogeneity of the aetiologies of cardiogenic shock and the small number of patients included, it is challenging to draw definitive conclusions about ECPELLA support. The indication for ECPELLA support is discussed in our multidisciplinary team and requires an individualised assessment. Furthermore, there is still no randomised evidence showing the benefit of adding LV unloading to VA-ECMO support, but VA-ECMO alone does not improve mortality in patients with acute coronary syndrome complicated by cardiogenic shock [21].

## Conclusions

According to our registry data, providing ECPELLA support could act mainly as a bridge-to-decision for patients experiencing severe refractory cardiogenic shock with lower 30-day mortality compared to previous cohorts. However, it comes at the cost of a significant number of complications. Further studies are needed to evaluate the efficacy and safety of ECPELLA and to identify optimal patient selection criteria.

### Supplementary Information

**Table S1** Detailed clinical profile of patients supported with ECPELLA
