# Supplementary material for: ECPELLA as a bridge-to-decision in refractory cardiogenic shock: a single-centre experience
Source: Neth Heart J. 2024 May 7;32(6):245–53. doi: 10.1007/s12471-024-01872-w (PMC11143097; doi:10.1007/s12471-024-01872-w)
Supplement: Supplementary file 1 — Table S1 Detailed clinical profile of patients supported with ECPELLA [file 12471_2024_1872_MOESM1_ESM.docx]

**Table S1** Detailed clinical profile of patients supported with ECPELLA

| **Case** | **Age** | **Gender** | **Cardiomyopathy** | **SCAI** | **ECPELLA order** | **Timing add-on support** | **Indication ECPELLA** | **Duration**  **support** | **Complications** | **Kidney failure** | **LVAD** | **30/90 day mortality** |
| --- | --- | --- | --- | --- | --- | --- | --- | --- | --- | --- | --- | --- |
| 1 | 56 | Female | Myocarditis | D | VA-EMCO first | IABP, late | Unloading^a^ | 160 |  | 0 | 0 | 0/0 |
| 2 | 62 | Female | Post-HTx | D | VA-EMCO first | late | Unloading^b^ | 192 | Haemothorax, access site bleeding | 1 | 0 | 0/1 |
| 3 | 68 | Male | ACS | D | Simultaneous | n/a | Severe shock | 216 | Bleeding: GI, access site  limb ischaemia  haemolysis | 1 | 0 | 1/1 |
| 4 | 36 | Male | Chronic | D | Simultaneous | n/a | Severe shock | 117 | Haemolysis | 1 | 1 | 0/0 |
| 5 | 34 | Male | Feochromocytoma | E | Simultaneous | n/a | Severe shock | 80 | Hb drop >3 mmol/l  haemolsyis | 1 | 0 | 0/0 |
| 6 | 54 | Male | ACS | D | Simultaneous | n/a | Severe shock | 262 | Haemolysis | 1 | 1 | 0/0 |
| 7 | 47 | Male | ACS | E | Impella first | early | Severe shock | 168 | Bleeding: pulmonary, GI  thrombus Impella | 1 | 0 | 1/1 |
| 8 | 41 | Male | Chronic | D | Simultaneous | n/a | Severe shock, high risk PCI | 92 |  | 0 | 0 | 0/0 |
| 9 | 45 | Female | Chronic | D | Simultaneous | n/a | Severe shock | 168 |  | 0 | 1 | 0/0 |
| 10 | 47 | Male | Myocarditis | D | Simultaneous | n/a | Severe shock | 240 |  | 1 | 1 | 0/1 |
| 11 | 43 | Male | Chronic | E | Simultaneous | n/a | Severe shock | 48 |  | 0 | 0 | 1/1 |
| 12 | 47 | Male | ACS | E | VA-EMCO first | IABP, late | Unloading^c^ | 128 | Limb ischaemia | 0 | 0 | 1/1 |
| 13 | 59 | Male | ACS | D | Simultaneous | n/a | Severe shock | 356 | Haemolysis  limb ischaemia  thrombus Impella | 0 | 1 | 0/0 |
| 14 | 54 | Female | Chronic | D | Simultaneous | n/a | Severe shock | 130 | Bleeding access site | 0 | 1 | 0/0 |
| 15 | 64 | Male | ACS | E | Impella first | late | Severe shock | 40 |  | 0 | 1 | 0/0 |
| 16 | 53 | Male | ACS | D | Simultaneous | n/a | Severe shock | 192 | Bleeding: haemothorax | 1 | 1 | 0/0 |
| 17 | 61 | Male | ACS | C | VA-EMCO first | late | Severe shock | 36 |  | 1 | 1 | 0/0 |
| 18 | 50 | Female | Chronic | D | Simultaneous | n/a | Severe shock | 117 | Hb drop >3 mmol/l | 0 | 1 | 0/0 |
| 19 | 60 | Male | ACS | E | Impella first | early | Severe shock | 190 | Access site dissection  Hb drop >3 mmol/l | 0 | 0 | 0/- |
| 20 | 45 | Female | Myocarditis | E | VA-EMCO first | IABP, late | Unloading^c^ | 260 | Bleeding removal VA-EMCO  papillary rupture -> MVR | 1 | 0 | 0/- |

^a^Pulmonary oedema and LV dilatation

^b^LV dilatation, aortic valve not opening

^c^High wedge pressure, severe LV dysfunction
